# Change Over 11–13 Year Periods in Quality of Life, Emotional Problems and Negative Stressful Life Events Among 13–17 Year Old Students

**DOI:** 10.1007/s10578-022-01325-8

**Published:** 2022-03-26

**Authors:** Thomas Jozefiak, Jan L. Wallander, Stian Lydersen

**Affiliations:** 1 Regional Centre for Child and Youth Mental Health and Child Welfare (RKBU Central Norway), Department of Mental Health, Norwegian University of Science and Technology, Trondheim, Norway; 2 Psychological Sciences and Health Sciences Research Institute, University of California, Merced, USA; 3 Regional Centre for Child and Youth Mental Health and Child Welfare (RKBU Central Norway), Department of Mental Health, Norwegian University of Science and Technology, MTFS, Pb 8905, 7491 Trondheim, Norway

**Keywords:** Changes of Quality of life, Adolescents, Emotional problems, Negative stressful life events

## Abstract

Studies investigating changes in the general population over time concerning adolescent self-reported Quality of life (QoL) are sparse. The aim of this study is to investigate stability and change over more than a decade in self-reported QoL, emotional problems, and negative stressful life-events among students. Three large cross-sectional samples (N = 1032, 4744 and 3826) of 13–17-year-old adolescents attending public school in the Norwegian County of Trøndelag provide data, one from 2017 to 2019 and two from 11 and 13 years earlier. We analyzed linear and binary linear regression adjusted for age. We found few indications of large changes in overall QoL. The exception was a 50% increase in reported emotional problems in both girls and boys. Girls also reported an increase of sexually uncomfortable/abusive acts from peers from 3.7 to 7.0%. The observed changes must be addressed through public health interventions targeting school as an important arena.

## Introduction

A common notion is that mental health problems are more prevalent in youth and they experience more stress today compared to, usually an undefined, earlier time. Indeed, seven in 10 adolescents in the U.S. see mental health problems as a major issue among their peers, including depression, anxiety, and suicidal behavior [1]. Empirical findings are less clear whether there have been significant changes over time, depending on factors such as when comparisons are made, where, and in what specific aspects of adolescent functioning.

For example, an earlier meta-analysis showed that there was no evidence for increased prevalence of child and adolescent depression over 30 years, between 1965 and 1996, in the USA [2]. More specifically, repeated assessment of emotional and behavioral problems among children and adolescents aged 7–16 in the U.S. general population from 1976 to 1999 showed an increase of problems in 1989 compared with 1976, but a decrease 10 years later, by 1999 [3]. A Dutch study found no evidence for a clear increase in problems in a 10-year period between 1983 to 1993 in children and adolescents from the general population [4]. A 16-year comparison between 1991 and 2007 found a significant decrease of parent-reported total emotional and behavior problems in 7–9 years old Norwegian children [5]. In contrast, studies from Scotland [6] using youth self-report from 1987, 1999 and 2006, and of English teenagers in 1986 and 2006 [7] showed a substantial increase in symptoms of anxiety and depression.

More recent findings from large-scale studies provide indications of increased prevalence of emotional problems in adolescents. For example, increases in anxiety and depressive problems were observed in teen-age girls from 2010 to 2019, and in boys from 2015 to 2019, based on representative Norwegian cross-sectional data sets, comprising 393,100 junior high school students [8]. Moreover, rates of major depressive episode in the past 12 months among over 600,000 adolescents aged 12 to 17 increased by 52% from 2005 to 2017 according to U.S. population survey data [9]. Nonetheless, these contradictory international findings over time raise the question anew whether children and adolescents have experienced a decrease in their mental health in the recent past.

Furthermore, mental health is a complex phenomenon [10], and changes of prevalence over time are also related to type of diagnosis. Moreover, depressive and anxiety *problems or symptoms* are not identical with depression or anxiety as a clinical *diagnosis*, as specified by international diagnostic systems. Psychiatric diagnoses are best obtained in operationalized interviews (for example the Child and Adolescent Psychiatric Assessment), that focus on ensuring that respondents understand what is being asked and on clearly defining levels of symptom severity, onset and duration of symptoms, and functional impairment [11].

Considering changes in adolescent functioning more broadly should include an examination of their quality of life (QoL). Acknowledging there are a number of definitions [12], here we define QoL as the adolescent’s perceived subjective well-being and satisfaction with life that is best evaluated by the adolescent, according to his/her own experience with regard to several life domains [13]. That is, QoL addresses perceived well-being in domains such as physical, psychological, and social functioning as well in the family, school, and leisure time for adolescents [12]. QoL can be distinguished from emotional and/or behavioral mental health problems, symptoms or disorders [14]. However, we are not aware of studies comparing QoL in adolescent cohorts across longer periods. Although not differentiated by age, data from the Eurobarometer for life satisfaction, which is related to QoL, show this generally to have improved between 2000 and 2016 in the general population in European countries [15]. If generalized to adolescents specifically, this would suggest a trend counter to reported data on mental health problems.

Negative stressful life events (SLE) have a known influence on emotional problems and QoL [16, 17]. Among the most salient stressful negative life events are experiencing or witnessing violence or sexual abuse [18], which may lead to impaired mental health in adolescence [19, 20], as well as later in life [21, 22]. Moreover, major changes in the family environment can be common stressful experiences in adolescence [23]. It is unclear to what extent experiencing stressful negative life events have changed in the past decades, but research suggests there has been a decrease for example in perpetrating physical violence [24]. The question remains whether this trend applies across most stressful negative life events?

Existing evidence underlines that there are important gender differences regarding emotional problems and QoL in adolescence. Girls report a higher level of emotional problems as well as more stress at school [8]. As well, girls reported lower QoL than boys in general population surveys [25–27]. It has also been reported that QoL decreases [25, 28] while emotional problems increase [9] during adolescence. Therefore, changes of emotional problems and QoL should be examined separately for boys and girls while controlling for age.

It will be valuable to examine whether QoL, mental health, and negative SLE reported by adolescents in the general population have changed over a recent decade. This information is important for optimizing community and special health services for adolescents. QoL assessment can be used in planning of therapeutic interventions [29]. If specific QoL-related life domains were detected as more problematic over time in the general population of adolescents, clinicians could screen their patients on such areas, beyond the actual reason for referral. In case of positive screening findings, preventive care for individuals who are at risk could be initiated before problems develop into severe psychopathology. Because QoL and depressiveness are negatively correlated [30], the same rationale can be applied to emotional problems. Early clinical interventions could prevent emotional problems from developing into psychiatric diseases, such as major depressive disorder or anxiety disorder. Many victims of violence, abuse, and other adversities in childhood or adolescence, develop serious mental and physical disease [31, 32]. Therefore, it is important to know about changes in prevalence of such negative life events in the adolescent population. This can be valuable information for individual clinicians, but also for constructing prevention programs. Planning prevention programs and future clinical services to benefit adolescents should be based on empirical evidence. Data from community-based surveys should provide the best evidence of changes.

Using data obtained from three samples in a county in Norway, we aimed to address the following questions in this research: Have there been changes in adolescents’ reported QoL, emotional problems of depression and anxiety, and negative life events during a decade ending in 2017–2019? Do any such changes differ by sex?

## Methods

### Participants and Settings

**Fig. 1:**
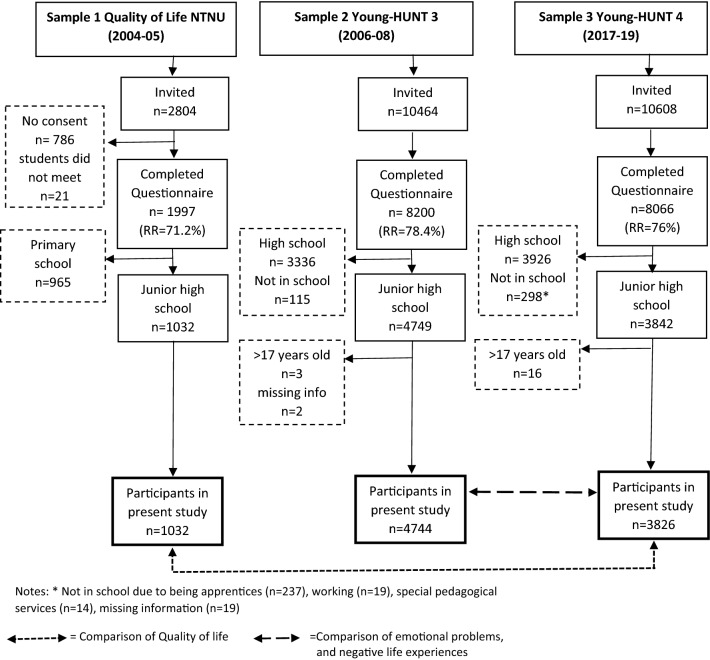
Flowchart of participants in present study

Three large cross-sectional samples of adolescents, age 12 to 17, attending public schools in the County of Trøndelag, Norway, provide data. One sample provides information from recent times and the other two from 11 and 13 years earlier, respectively (see Table 1; Fig. 1). The county contains 8.7% (464,060 inhabitants) of Norway’s population and is situated in its center covering 44,267 km^2^. The population is ethnically and socioeconomically homogeneous. The largest town, Trondheim, is situated in the southern area, comprising 42.3% of the county total population. The rest of the population reside in both rural and smaller urban areas. The county is fairly representative of Norway as a whole regarding geography, economy, education, industry, income, age distribution, morbidity, and mortality [33–36].

**Table 1:** Sample characteristics of the adolescents participating in the study

	Sample 1				Sample 2				Sample 3			
	n	M	SD	Range	n	M	SD	Range	n	M	SD	Range
Sex												
Male	511				2355				1946			
Female	521				2389				1880			
Total	1032				4744				3826			
Age (y)		14.1	1.0	12–17		14.6	0.9	13–17		14.5	0.9	13–17
SES^a^	–				4327	2.08	0.49	1–3	3765	2.13	0.48	1–3
Study	“Quality of Life-NTNU”					Young-HUNT3 Survey				Young-HUNT4 Survey		
Time of data-collection	2004–2005					2006–2008				2017–2019		
Response rate	71,3% (for total sample 8–17 years)					78.4% (for total sample 13 to 19 years)				76.0% (for total sample 13 to 19 years)		

^a^“Is your family better or worse off economically than others?” (1 = worse off, 2 = the same, 3 = better off)

#### Sample 1 (2004–2005)

The “Quality of Life and Mental Health—NTNU” survey was conducted in 2004–2005 [26] in the southern area of the county. Representation was obtained by using a cluster-sampling procedure of all public schools. Of 426 grade cohorts (defined as the students attending a specific grade at a single school), 61 were randomly selected for the study at 51 schools. Grade cohorts had been stratified with regard to gender and geography. Of the primary and junior high school students, ages 8–17, who were invited, 1997 participated. Among these were 1032 attending either 8th or 10th grade available for the present study.

#### Sample 2 (2006–2008)

The Young-HUNT3 Survey, the adolescent part of HUNT, “The Trøndelag Health Study, Norway” [33], was conducted in 2006–2008 in the northern area of the county, and included adolescents ages 13–19. Adolescents attending 1 of 66 junior high or high schools, or no school, were invited to complete the survey, of which 8200 did. Of the 4749 attending junior high school, 4744 provided valid questionnaires for the present study.

#### Sample 3 (2017–2019)

The Young-HUNT4 Survey [37] was conducted in 2017–2019 in the same manner as the Young-HUNT3 Survey. From the 8066 participants across the full age range, 3826 junior high school students provided valid questionnaires for the present study.

### Assessment Procedure

*Sample 1* data were collected in schools. One teacher at each school informed the students about the project and sent an information letter to their parents. A researcher was present at each school when the students completed the questionnaires. Students completed the survey on their own, but for those with reading problems questions were read aloud. The questionnaires were marked with an ID number, sealed in an envelope, and placed in a collection box by individual students. Those not present the day of data collection completed their questionnaires individually during the following week. Further details are provided elsewhere, [26].

*Sample 2* data were collected during class time. Students were informed about the project and an information letter was sent to their parents. Students completed the survey on their own, but for those with reading problems questions were read aloud. The questionnaire was sealed in an envelope and placed in a collection box by individual students. Adolescents temporarily away from school on the day of survey completion received the survey about 1 month later. Further details are provided elsewhere, [33].

*Sample 3* was administered the survey during a class time with a procedure highly similar to that for Sample 2.

### Measures

QoL was measured in Sample 1 and 3 using the Inventory of Life Quality in Children and Adolescents (ILC) [38, 39]. This 7-item self-report questionnaire includes one item for global evaluation of QoL and six items that address school, perceived relationship with family and friends, the ability to activate themselves with hobbies and interests when alone, and physical and mental health. Each item is rated on a 5-point Likert scale, for example “How do you cope with work at school?” where 1 = “very good”, 5 = “very bad”. A QoL LQ_0–28_ sum scale is calculated across all items, with higher scores reflecting higher QoL. Further, a Problem score PR_07_ can be calculated by counting the number of items rated 3 = “neither good nor bad” and above, indicating the number of domains with a problem. This Norwegian version shows good internal consistency (α = 0.80–0.81) and 2-week test–retest reliability (ICC = 0.86). The validity of the ILC is satisfactory based on empirical factor structure matching expectations and convergent and discriminative validity compared with gold standard QoL and depressive symptoms instruments [38].

*Emotional problems of depression and anxiety* were measured in Sample 2 and 3 using the Hopkins Symptom Checklist-5 (SCL-5), [40, 41] which consists of five items from the 25-item version, addressing for example “Felt down and sad”. The SCL-5 has very high correlation (r = 0.92) with the SCL-25 and a satisfactory reliability [40]. Responses are provided on a four-point scale (1 = “not bothered”, 4 = “very bothered”), from which a mean score is calculated. Whereas a mean score < 2 is indicating no emotional problems, a mean score ≥ 2 is indicating symptoms having mild, moderate or severe impact on the respondent.

*Negative stressful life-events* were measured in Samples 2 and 3 with eight items addressing occurrence of serious disease in family, death of a close person, a catastrophic serious accident, victimization of physical violence, witness of physical violence, sexually uncomfortable/abusive acts from peers and, separately, from adults, (see Table 3 for complete survey questions). Responses were obtained using similar but not identical 3-point scales (“No/never”, “Yes during last year/once,” “Yes, during lifetime/several times”). Therefore, each item was scored as 0 if answered “No/Never” and 1 if answered either of the other two responses, and a Total scale (score 0–8) was summed, indicating the number of negative life events reported.

*Socio-economic status* (SES) was measured in Samples 2 and 3 using the question whether they felt his or her family is better or worse off economically than others. With response choices being 1 = worse off, 2 = the same, 3 = better off).

*Age and sex* Participants self-reported age and sex.

### Statistical Analysis

Missing values for the measures ranged from 2.8 to 8.8%. These were imputed using the expectation maximization algorithm for ILC and SCL-5. For SES and Negative SLE, available case analysis was used.

We compared proportions using binary linear regression adjusted for age. For scale variables we used linear regression adjusted for age, except for comparison of SES, where t-test was applied. Due to the non-normality of the ILC variables, these analyses were carried out with bootstrapping, using B = 1000 bootstrap replications and BCa (Bias corrected and accelerated method). All analyses were carried out separately for girls and boys. Two-sided *p *values < 0.05 indicated statistical significance, but due to multiple hypotheses, *p* values between .01 and .05 should be interpreted with caution. Binary linear regressions were carried out in Stata 16, and the other analyses in SPSS 26.

### Ethics

Written information was provided, and informed consent obtained from adolescents and at least one parent for each prior to inclusion in each Sample.

The Norwegian Ethical Committee for Medical Research and the Norwegian Social Science Data Services approved the studies (REK registration no. for Young-HUNT3 is 4.2006.250, 06.04.2006, Concession no. for Young-HUNT4 is 17/00426-7/GRA) and REK registration no. for “QoL & Mental Health NTNU project” is 140-02.)

## Results

### Socio-economic Status

Adolescents in Sample 3 rated their SES as significantly higher in 2017–2019 compared to those in Sample 2 in 2006–2008 (*M* = 2.13; *SD* = 0.49; n = 4327 vs. *M* = 2.08; *SD* = 0.48; n = 3765, *t*(7983) = − 4.49; *P* < .001). However, because Pearson correlation between QoL (ILC LQ_0–28_) and SES in Sample 3 was low, *r* = 0.16; *P* = .01, n = 3826 and n = 3765, respectively), we decided not to adjust for SES in statistical analyses.

### Quality of Life (ILC)

Table 2 shows comparisons in QoL between Samples 1 and 3 for the 7 ILC items. There were no significant differences in overall QoL during the 13–14-year period from 2004–2005 to 2017–2019, based on the ILC LQ_0–28_ sum scores for neither girls nor boys. Likewise, there were no significant difference in the number of life domains that were reported to be problematic (PR_07_ scores) during this period.

**Table 2:** Comparison of quality of life reported between 2004–2005 and 2017–2019 for girls and boys based on ILC domains and scores

ILC domains and scores	Possible score range	Girls				Boys			
		2004–2005 Sample 1 (n = 511)	2017–2019 Sample 3 (n = 1946)	Mean difference (95% CI)	p value	2004–2005 Sample 1 (n = 521)	2017–2019 Sample 3 (n = 1880)	Mean difference (95% CI)	p value
		M (SD)	M (SD)			M (SD)	M (SD)		
ILC 1 School	1–5^a^	2.17 (0.89)	2.19 (0.89)	− 0.04 (− 0.12 to 0.05)	.40	**2.11** (0.82)	2.02 (0.84)	− 0.14 (− 0.22 to − 0.06)	**.002**
ILC 2 Family	1–5	**1.68** (0.86)	1.55 (0.75)	− 0.16 (− 0.24 to − 0.08)	**.001**	1.57 (0.79)	1.57 (0.73)	− 0.02 (− 0.09 to 0.07)	.71
ILC 3 Friends	1–5	1.61 (0.73)	1.66 (0.74)	0.03 (− 0.04 to 0.11)	.35	1.58 (0.71)	1.60 (0.70)	0.02 (− 0.05 to 0.09)	.54
ILC 4 Alone	1–5	1.85 (0.84)	1.90 (0.86)	0.02 (− 0.06 to 0.11)	.55	1.66 (0.77)	**1.79** (0.80)	0.13 (0.05 to 0.20)	.**004**
ILC 5 Physical health	1–5	2.11 (0.91)	2.09 (0.84)	− 0.06 (− 0.16 to 0.02)	.16	1.88 (0.92)	1.89 (0.83)	0.00 (− 0.09 to 0.09)	.95
ILC 6 Mental health	1–5	1.99 (0.89)	2.12 (0.98)	0.06 (− 0.02 to 1.14)	.18	1.68 (0.79)	**1.80** (0.83)	0.11 (0.02 to 0.19)	**.010**
ILC 7 Global QOL	1–5	2.01 (0.87)	2.03 (0.93)	− 0.04 (− 0.13 to 0.06)	.42	1.70 (0.71)	1.74 (0.76)	0.02 (− 0.05 to 0.09)	.60
PR_0–7_	0–7^a^	1.64 (1.83)	1.66 (1.91)	− 0.12 (− 0.31 to 0.06)	.20	1.07 (1.45)	1.10 (1.67)	− 0.03 (− 0.19 to 0.11)	.69
LQ_0–28_	0–28^b^	21.59 (4.06)	21.47 (4.41)	0.18 (− 0.23 to 0.59)	.40	22.82 (3.75)	22.59 (4.10)	− 0.12 (− 0.49 to 0.27)	.53

**Bold** = significant *p *value and higher mean, *QOL* quality of life, *PR* problem score, *LQ* life quality score

^a^Higher score indicates poorer QoL. ^b^Higher score indicates better QoL

A more detailed analysis of the seven ILC domains revealed for girls a significantly (*P* = .001) increased QoL over this period related to the family only (see Table 2). Boys reported significantly (*P* = .002) increased school functioning, but decreased ability to activate themselves with hobbies and interests when *alone* (*P* = .004) as well as mental health during this period.

### Emotional Problems of Depression and Anxiety

Comparing Samples 2 and 3, 22.8% of girls reported mild, moderate, or severe impact of emotional problems in 2006–2008, which increased to 34.6% 11 years later in 2017–2019 (*P* < .001). For boys, 8.6% reported mild, moderate, or severe impact of emotional problems in 2006–2008, which 11 years later had significantly increased to 12.6%, (*P* < .001).

### Negative Stressful Life Events

Table 3 shows comparisons between Samples 2 and 3 representing the 11-year period from 2006–2008 and 2017–2019, for eight negative SLE and total score, separately for girls and boys. The total number of negative SLE increased slightly but significantly for both girls and boys. Both sexes reported significantly increased serious disease in the family and serious accident, whereas being victim or witness of physical violence decreased in this period for both sexes. Boys, but not girls, reported significant increase of death of a close person and decrease of sexually uncomfortable/ abusive acts from adults. Girls reported a significantly increase in sexually uncomfortable/ abusive acts from peers during the 11-year period, which boys did not.

**Table 3:** Comparison of negative life events between 2006–2008 and 2017–2019 for girls and boys

Negative life events Have any of the following things happened to you?	Girls				Boys			
	2006–2008 Sample 2	2017–2019 Sample 3	Mean difference (95% CI)^a^	p^a^	2006–2008 Sample 2	2017–2019 Sample 3	Mean difference (95% CI)^a^	p^a^
	% (Proportion)	% (Proportion)			% (Proportion)	% (Proportion)		
That you or someone in your family has been **seriously ill**	54.6 (1233/2259)	**59.6** (1124/1886)	**5.6 (2.6 to 8.6)**	** < .001**	42.2 (934/2213)	**50.9** (916/1800)	**9.5 (6.4 to 12.6)**	** < .001**
**Death** of a loved one	63.0 (1421/2254)	64.8 (1222/1887)	2.0 (− 1.0 to 4.9)	.19	55.0 (1216/2211)	**61.9** (1115/1800)	**7.7 (4.6 to 10.7)**	** < .001**
A **catastrophe** (fire, hurricane, etc.)	8.3 (188/2259)	8.7 (165/1891)	0.6 (− 1.1 to 2.3)	.49	9.2 (204/2219)	9.6 (172/1797)	0.8 (− 1.0 to 2.6)	.37
A **serious accident** (e.g. a very serious car accident)	12.3 (278/2255)	**14.4** (271/1887)	2.2 (0.1 to 4.3)	**.037**	11.3 (251/2218)	**14.2** (254/1793)	**3.2 (1.1 to 5.2)**	**.003**
**Subjected to violence** (beaten/injured) by someone close to me	**5.7** (128/2260)	4.0 (75/1888)	− **1.7 (**− **3.0 to **− **0.4)**	**.011**	**9.2** (205/2219)	3.4 (62/1800)	− **5.8 (**− **7.3 to **− **4.3)**	** < .001**
**Seen others violently hurt**	**11.9** (269/2263)	8.7 (163/1883)	− **2.3 (**− **4.0 to **− **0.5)**	**.012**	**20.1** (447/2219)	15.0 (270/1799)	− **3.4 (**− **5.7 to **− **1.1)**	**.004**
Been **subjected to sexually uncomfortable/abusive acts** by someone about your age	3.7 (84/2256)	**7.0** (131/1882)	**3.0 (1.6 to 4.3)**	** < .001**	2.1 (46/2218)	1.6 (28/1794)	− 0.4 (− 1.2 to 0.4)	.34
Been **subjected to sexually uncomfortable/abusive acts** by an **adult**	2.5 (56/2254)	3.2 (60/1878)	0.8 (− 0.2 to 0.18)	.099	**1.7** (38/2219)	0.9 (16/1788)	− **0.7 (**− **1.4 to **− **0.04)**	**.038**
NLE total *M* (*SD*) N	1.61 (1.27) 2206	**1.70** (1.27) 1836	**0.11 (0.03 to 0.19)**	**.005**^**b**^	1.50 (1.44) 2167	**1.58** (1.24) 1768	**0.11 (0.03 to 2.0)**	.**009**^b^

^a^From binary linear regression adjusted for age ^b^From linear regression adjusted for age **Bold** = significant *p* value and higher mean

## Discussion

Results show few indications of large changes in overall QoL or total negative SLE reported by adolescents across 11- or 13-year periods ending in 2017–2019. The exception was a 50% increase over the 11-year period in reporting emotional problems in both girls and boys and for girls an almost doubling in reported sexually uncomfortable/abusive acts from peers.

### Quality of Life (ILC)

There was overall a balance in changes across life domains and few specific changes. In 2017–2019, compared to 13 years earlier, girls reported better QoL related to their families, while boys did for school. Boys’ ability to activate themselves when alone, however, decreased during this period as did their mental health, but there was no change for girls.

Regarding the QoL dimension School, in contrast to our results, girls in Australia reported a decrease in HRQOL. However, in Norway reports [42] showed that girls stress related to school increased from 3% in the 6th grade to 30% in the 10th grade, while 51% of girls in the 6th grade reported that they liked school very much vs. only 35% who did in the 10th grade. In our study, boys increased their school-related QoL significantly during the 13-year period. This is interesting, because In Norway, girls achieve better grades than boys from junior high school throughout their remaining education period [43]. Thus, even though boys perform more poorly in school, their experience in school appears to have improved in recent times.

### Emotional Problems of Depression and Anxiety

Regarding the QoL domain “Mental health”, boys reported a significant decrease, while girls did not. However, additional analyses of reported emotional problem based on the more targeted assessment confirmed that the proportion reporting at least mild problems increased by a factor of 50% in *both* girls and boys during the 11-years period ending in 2017–2019. Yet, as is well known, a considerably lower proportion of boys compared to girls reported emotional problems. Of course, mental health is a complex phenomenon [10]. While disorders like Attention Deficit Hyperactivity Disorder showed no systematic change in prevalence in epidemiological studies, there has been a substantial increase in affective problems in high-income countries, especially among adolescents [9, 10].

Epidemiological research from Greece, Germany, Sweden, Iceland, China and New Zealand indicates a long-term rise in self-reported emotional problems among adolescents from the 1980s onwards until the first decade of the twenty-first century [10]. In Norway, an increase in both male and female adolescent depressive problems from three nationwide representative surveys was detected during the 1990s, but no significant change between 2002 and 2010 [44]. There is relative sparse evidence yet of recent trends, and the results of the present study raise the question anew whether adolescents have experienced a worsening in their mental health in the more recent past. Our results show clear substantial increase in self-reported emotional problems of depression and anxiety from 2006–2008 to 2017–2019. This corresponds well to results of the Norwegian representative national survey 2010–2019 [8]. However, depressive and anxiety problems or symptoms are not identical with depression or anxiety as a clinical *diagnosis*. During the recent years there has been more public recognition and acceptance of mental health problems. Thus, we cannot exclude the possibility our observed trend regarding emotional problems rather represents one way that youth express experiences of more stress, for example related to school [8, 42] and parents’ [45] and peer ‘s expectations of “being a successful youth.” Meeting perceived expectations includes self-expression on social media. In any case, the observed changes should be of major concern.

### Negative Stressful Life Events (SLE)

Girls reported approximately a two-fold increased prevalence of sexually uncomfortable/abusive acts from peers while boys reported more than a 50% decrease in sexually uncomfortable/abusive acts from adults. This is an important disconcerting finding. Our results cannot disentangle whether this represents an actual increase in victimization of girls or an increased likelihood of recognizing in recent times such act as victimization when they occur. Moreover, to what extent is the observed substantial increase of emotional problems in girls linked to their higher reported level of sexually uncomfortable/abusive acts among girls? Research into this issue is much needed.

Rates of antisocial behavior appear to have levelled off since the 1990s in many high income countries [10, 24] and our findings concerning experiencing or witnessing violence are consistent with this trend. Prevalence for experiencing serious accidents increased for both sexes between 2006–2008 and 2017–2019. This, too, might be linked to the increase in reported emotional problems.

## Strengths and Limitations

The large sample sizes and representativeness regarding sex, age, and geography are strengths of the study. Even though Sample 1 was smaller, it was carefully stratified as a cluster sample and obtained a response rate of 71%. Samples 2 and 3, from Young-HUNT were larger, containing over 76% of the entire target population. Response rates for the three samples were satisfactory for this type of study. Nonetheless, 24–29% of eligible adolescents did not participate. The design and ethics approval of the studies did not allow for conducting any attrition analysis. We must assume that non-attendees display poorer outcomes on average. If this is the case, the present study would positively overestimate QoL and well-being of adolescents. However, it is unclear that this bias would influence the observed trends that is have a different impact at different periods.

Another limitation is the uneven time periods in different analyses, with the 13-year interval for QoL compared to the 11-year interval for the other outcomes. Possibly mitigating this, there were no larger changes in the Norwegian culture or society during the 2 years separating the time intervals. Furthermore, we only had access to a highly limited subjective measure of SES and no objective information about parent education and income. There is some evidence from adults that objective measures of SES are significantly associated with health outcomes [46], and that subjective social status may be an even better predictor, but no findings that we are aware of pertaining to our age group. However, the lack of objective SES information in our study represents a clear limitation.

## Summary

Contradictory findings in past and recent research raise the question anew whether adolescents have experienced a decrease in their adjustment and mental health in the recent past. The aim of this study was to investigate stability and change over more than a decade in self-reported QoL, emotional problems, and negative stressful life-events in adolescents. Three large cross-sectional samples (N = 1032, 4744 and 3826) of adolescents attending schools in one Norwegian county provide data, one from 2017–2019 and two from 11 and 13 years earlier, respectively. We analyzed linear and binary linear regression adjusted for age. We found few indications of large changes in overall QoL and total negative SLE reported by adolescents across 11- or 13-year periods ending in 2017–2019. The exception was a 50% increase in both girls and boys reporting emotional problems of depression and anxiety. Girls also reported an increase of sexually uncomfortable/abusive acts from peers from 3.7 to 7.0%. Research is needed to explain these observed substantial increasing trends in emotional problems and uncomfortable/abusive acts reported by girls. Clinicians in community as well as special health services for adolescents should be more aware of the possible emotional pressure and stress their adolescent patients are exposed to and offer preventive care for those at risk. Especially female patients should be actively asked if they have experienced uncomfortable/abusive acts. Last but not least, our results must be addressed through public interventions targeting school as an important arena where unhealthy stress is experienced. Interventions will need to focus on high expectations from adults and peers and sexual abuse. Fully-digitalized prevention programs to prevent sexual abuse and violence for the entire span of primary education is now available in Norway [47, 48].
